# Obstructive Sleep Apneas and Cardiovascular Diseases

**DOI:** 10.3390/clockssleep8020028

**Published:** 2026-05-21

**Authors:** Vincenzo Castiglione, Paolo Morfino, Iacopo Fabiani, Francesco Gentile, Edoardo Airò, Benedetta Volpi, Daniela Cardinale, Claudio Passino, Alberto Giannoni, Michele Emdin

**Affiliations:** 1 Health Science Interdisciplinary Research Center, Scuola Superiore Sant’Anna, 56127 Pisa, Italy; 2 Cardiology and Cardiovascular Medicine Division, Fondazione Monasterio, 56124 Pisa, Italy; 3 Cardioncology Unit, European Institute of Oncology, IRCCS, 20141 Milan, Italy

**Keywords:** obstructive sleep apnea, obesity, heart failure, continuous positive airway pressure

## Abstract

Obstructive sleep apnea (OSA) is a sleep-disordered breathing condition characterized by recurrent upper-airway obstruction, leading to intermittent hypoxemia, sleep fragmentation, and sympathetic activation. OSA is highly prevalent in patients with cardiovascular diseases and is strongly associated with hypertension, atrial fibrillation, coronary artery disease, heart failure, and adverse prognosis. This review summarizes current evidence on the pathophysiology of OSA, its cardiovascular consequences, and available diagnostic and therapeutic strategies, with particular attention to clinical implications in cardiology practice. We discuss established treatments such as lifestyle interventions, continuous positive airway pressure, mandibular advancement devices, and selected surgical options, as well as emerging therapies, including pharmacological approaches targeting weight loss and ventilatory control. While OSA treatment improves symptoms and quality of life, evidence for cardiovascular event reduction remains heterogeneous and appears strongly influenced by patient selection and treatment adherence. Identifying patients most likely to benefit from targeted OSA management remains a key challenge.

## 1. Introduction

Obstructive sleep apnea (OSA) is a form of sleep-disordered breathing (SDB) caused by partial or complete upper-airway obstruction during sleep, leading to repeated episodes of reduced (hypopnea) or absent (apnea) inspiratory flow despite persistent respiratory effort [1].

SDB includes respiratory events such as apneas and hypopneas, typically associated with desaturation and/or arousals during sleep. An apnea is defined as a ≥90% reduction in airflow for ≥10 s [2]. Hypopnea is variably defined as a ≥30% airflow reduction for ≥10 s, associated with either a ≥3% desaturation or arousal, or a ≥4% desaturation alone [2]. Both definitions are used in clinical studies, and their relative diagnostic impact remains under investigation [2].

OSA must be distinguished from central apneas (CAs), which result from impaired respiratory drive rather than upper-airway obstruction. CAs are linked to altered chemoreflex control, circulatory delay, and increased “plant gain”, i.e., ventilatory oversensitivity to CO_2_ changes [1,3,4]. A typical manifestation is Cheyne–Stokes breathing, with alternating CA and hyperventilation phases [3]. If these oscillations do not meet CA criteria, the pattern is termed periodic breathing, which can also be detected during exercise testing [5].

In OSA, airflow cessation occurs despite continued thoracoabdominal effort, whereas in CA both airflow and respiratory effort are absent due to a lack of central respiratory drive [1]. Both conditions may coexist in the same patient, and mixed apneas typically begin as central events and end with obstructive features [2].

OSA has major effects on quality of life and on multiple organs, particularly the cardiovascular system [6]. Typical symptoms include asthenia and excessive daytime sleepiness, leading to reduced attention, cognitive impairment, decreased productivity, and increased risk of work and road accidents [7]. OSA is associated with a higher incidence of type 2 diabetes, systemic and pulmonary hypertension (PH), coronary artery disease (CAD), atrial fibrillation (AF), stroke, heart failure (HF), and increased mortality [8].

**Figure 1:**
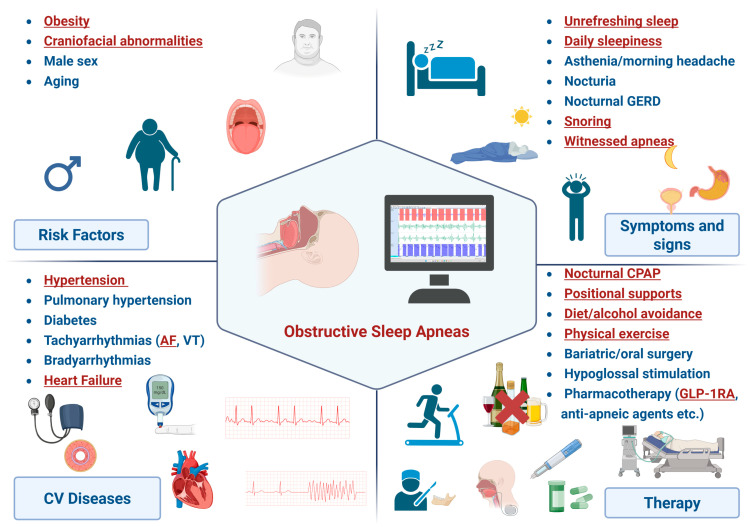
Relationship between cardiovascular diseases and obstructive sleep apneas. AF, atrial fibrillation; CPAP, continuous positive airway pressure; CV, cardiovascular; GERD, gastroesophageal reflux disease; GLP1-RA, glucagon-like peptide-1 receptor agonists; VT, ventricular tachycardia. Created in BioRender. Emdin, M. (2026) https://BioRender.com/jag2yxy.

Despite its high prevalence in cardiovascular patients and clear prognostic impact, OSA remains underdiagnosed and undertreated in cardiology. This narrative review summarizes current evidence on OSA, with emphasis on its interaction with cardiovascular disease and therapeutic implications (Figure 1).

## 2. Staging

OSA severity is primarily assessed through the apnea–hypopnea index (AHI), defined as the number of apneas and hypopneas per hour of sleep [1]. Home monitoring devices lack electroencephalographic monitoring (EEG) and therefore estimate sleep time; in this setting, AHI is reported as the respiratory event index (REI) [1,9]. OSA severity is graded as follows: AHI/REI < 5 (normal), 5–15 (mild), 15–30 (moderate), >30 (severe) [2]. When polysomnography is available, some centres report the respiratory disturbance index (RDI), which also includes respiratory-effort-related arousals (RERAs), defined as sleep interruptions that do not formally meet the criteria for apnea or hypopnea, but still disturb breathing during sleep and may trigger arousal [10]. Desaturation-based metrics are also used in addition to event-based indices. The oxygen desaturation index (ODI) counts ≥3% desaturation events per sleep hour [2], but T < 90% (time spent with SaO_2_ < 90%) is often more informative [2,11].

## 3. Epidemiology

OSA is the most common sleep-related respiratory disorder, with comparable prevalence across industrialized countries [8]. In the Wisconsin Sleep Cohort, mild OSA (AHI 5–15) affected 34% of men and 17% of women aged 30–70, while moderate–severe OSA (AHI > 15) occurred in 13% of men and 6% of women [12]. However, OSA remains largely underdiagnosed, with an estimated 24 million undetected cases in the United States alone [13]. Between 1990 and 2010, OSA prevalence increased by ~30%, rising by 4% in women and 8% in men [12]. Prevalence rises with age and is roughly twice as high in men as in women, although it increases in women after menopause [12]. OSA is strongly correlated with overweight and obesity, with prevalence increasing proportionally to body mass index (BMI) [12]. Among patients with cardiovascular disease, OSA prevalence reaches ~40%, markedly higher than in the general population [14].

### Cardiovascular Patients at High Risk for OSA

Certain patient subgroups carry a particularly high probability of clinically significant disease and should therefore be prioritized for targeted screening.

Patients with resistant hypertension represent one of the highest-risk groups, as OSA frequently contributes to poor blood pressure control through sympathetic activation and nocturnal fluid redistribution [15]. Similarly, OSA is highly prevalent in patients with atrial fibrillation, particularly in those with recurrent arrhythmia after cardioversion or catheter ablation [16]. Patients with heart failure also warrant focused attention, especially those with preserved ejection fraction, obesity-related phenotypes, or persistent congestion, in whom SDB is common and often underrecognized [17].

Additional high-risk categories include patients with pulmonary hypertension, coronary artery disease with recurrent ischemic events, and prior stroke or transient ischemic attack [18].

## 4. Pathophysiology

### 4.1. Anatomical Mechanisms

Airway patency during sleep depends on the activity of upper-airway dilator muscles (e.g., the genioglossus and soft-palate elevator muscles), which prevent tongue collapse and pharyngeal narrowing [19]. Patients with OSA often have structurally narrow upper airways due to parapharyngeal fat deposition, fatty infiltration of the pharyngeal muscles, or craniofacial abnormalities. The severity of OSA may vary across individuals and populations because of differences in the prevalence of soft-tissue abnormalities (more common in Western countries) and craniofacial skeletal abnormalities (more common in Eastern countries). The normal reduction in dilator muscle tone during sleep promotes airway collapse in anatomically predisposed individuals [19].

### 4.2. Functional Mechanisms

Beyond structural collapse, OSA is influenced by a low arousal threshold, reduced compensatory activation of upper-airway muscles, and unstable ventilatory control (“high loop gain,” i.e., an exaggerated chemoreflex response to changes in blood gases) [20,21]. The balance among these traits contributes to distinct OSA phenotypes. High loop gain also contributes to CA, explaining the frequent coexistence of OSA and CA or mixed apneas [22]. In rare cases, pharyngeal neuropathy or denervation may impair reflex activation of upper-airway dilator muscles [23]. Hormonal factors such as leptin may modulate dilator muscle tone, possibly explaining why equally obese individuals exhibit different susceptibility to OSA [24].

### 4.3. Impact of OSA on Cardiovascular Pathophysiology and Metabolism

Intermittent hypoxia, hypercapnia, and intrathoracic pressure swings during OSA trigger repeated arousals and sleep fragmentation, which are major contributors to daytime sleepiness [19]. Hypoxia and hypercapnia stimulate peripheral chemoreceptors, while recurrent arousals enhance central sympathetic outflow, resulting in persistent activation of the sympathetic nervous system. This neurohumoral response increases heart rate, peripheral vasoconstriction, and blood pressure (BP), with effects that may extend into wakefulness [25]. OSA therefore causes nocturnal BP surges and a non-dipping or reverse-dipping profile, which may persist during the daytime because of sustained sympathetic activation [26]. Indeed, OSA represents one of the main causes of nocturnal and resistant hypertension [27].

Sympathetic overactivity, together with intermittent hypoxia and reduced intrathoracic pressure, may also activate the renin–angiotensin–aldosterone system (RAAS) through renal vasoconstriction, reduced renal perfusion, and enhanced renin release. Increased angiotensin II and aldosterone levels promote sodium retention, nocturnal rostral fluid shift, vasoconstriction, oxidative stress, and vascular remodelling [28]. Fluid accumulation within peripharyngeal tissues may narrow the upper-airway lumen and increase tissue pressure, thereby further amplifying upper-airway collapsibility [29].

**Figure 2:**
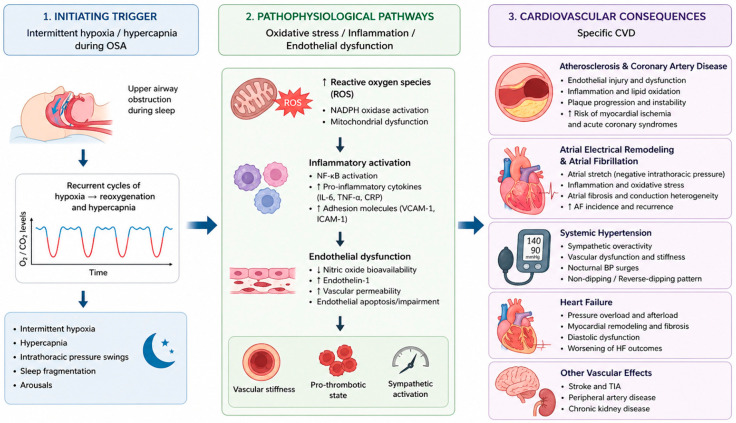
Pathophysiological cascade linking obstructive sleep apnea (OSA) to cardiovascular disease (CVD). IL-6, interleukin-6; TNF-α, tumour necrosis factor-α; CRP, C-reactive protein; VCAM-1, vascular cell adhesion molecule-1; ICAM-1, intercellular adhesion molecule-1; NF-κB, nuclear factor kappa B; AF, atrial fibrillation; TIA, transient ischemic attack.

Beyond their hemodynamic effects, recurrent cycles of intermittent hypoxia followed by reoxygenation promote oxidative stress, systemic inflammation, and endothelial dysfunction, thereby accelerating vascular injury. These processes are mediated in part by the generation of reactive oxygen species and upregulation of pro-inflammatory pathways involving hypoxia-inducible factor-1, nuclear factor κB, endothelin-1, interleukins, tumour necrosis factor-α, and interferon-γ. The resulting reduction in nitric oxide bioavailability, increased arterial stiffness, platelet activation, and impaired endothelial repair contribute to the development and progression of hypertension and atherosclerosis [30] (Figure 2).

These mechanisms are also relevant to coronary artery disease. Chronic inflammation, oxidative stress, platelet activation, and surges in sympathetic tone may promote plaque progression and instability, increasing the risk of myocardial ischemia and acute coronary syndromes. In parallel, repetitive negative intrathoracic pressure swings increase myocardial wall stress and oxygen demand, further exacerbating ischemic burden in susceptible individuals [31]. OSA also contributes to cardiac electrical and structural remodelling. Sympathetic activation, atrial stretch related to negative intrathoracic pressure, and inflammation may favour atrial fibrosis and conduction heterogeneity, thereby increasing susceptibility to atrial fibrillation and arrhythmia recurrence after rhythm-control strategies [32]. In addition, sympathetic overactivation and catecholamine excess reduce insulin sensitivity and may induce β-cell apoptosis, thereby linking OSA to diabetes mellitus [33,34].

## 5. Clinical Presentation and Diagnosis

Despite being common, OSA is rarely reported spontaneously, so screening relies on recognizing suggestive symptoms and signs. Restless, non-restorative sleep and excessive daytime sleepiness are the most frequent symptoms, often accompanied by fatigue, morning headache, nocturia, and nocturnal reflux [35,36]. Less commonly, during a nocturnal awakening, patients may complain of a sensation of choking, suffocation, or palpitations [8]. Snoring is common (50–60% of cases) but nonspecific, while witnessed apneas are more specific though less frequently reported (10–15%) [37,38,39].

The Epworth sleepiness scale (ESS) is widely used to quantify sleepiness, although it is not specific to OSA [40]. Screening tools such as the Berlin and STOP-Bang questionnaires help estimate OSA risk in primary care and hospital settings (Table 1) [41,42]. Physical findings suggestive of OSA include obesity, large neck circumference, or upper-airway anatomical narrowing. Polysomnography remains the diagnostic gold standard, providing full sleep staging and respiratory monitoring (airflow, thoracoabdominal movements, oximetry, electrocardiography, EEG). Limitations include cost, limited availability, and possible “first-night effect” [43]. Home cardiorespiratory monitoring offers a more accessible alternative but lacks EEG and has slightly lower diagnostic accuracy (sensitivity: 79%, 95% confidence interval [CI]: 71–86%; specificity: 79%, 95% CI: 63–89%) [44,45]. Oximetry-only devices or implantable device sensors may assist in screening but still require confirmatory testing [46]. There is growing interest in the potential role of wearable devices (bracelets, chest bands, adhesive patches, headsets, and rings) for both the diagnosis and monitoring of OSA [47].

## 6. Obstructive Sleep Apneas and Cardiovascular Disease

### 6.1. Systemic Hypertension

Intermittent hypoxia in OSA activates the sympathetic system, increasing arterial tone and BP. OSA is found in 30–50% of hypertensive patients, and ~50% of individuals with OSA develop hypertension [48]. OSA represents the leading cause of resistant hypertension, accounting for up to 80% of cases [1,49]. Patients with untreated OSA have a 2–3-fold higher risk of developing hypertension compared with those receiving treatment [50,51,52]. A meta-analysis of 26 studies showed a dose–response relationship: mild, moderate, and severe OSA increased hypertension risk by 18%, 32%, and 56% respectively [52]. The association is stronger in Caucasian patients and in men compared with women [52,53]. Notably, greater night-to-night variability in AHI independently predicts uncontrolled hypertension, increasing risk by ~50–70% regardless of OSA severity [54]. OSA is highly prevalent in non-dipper (71%) and reverse-dipper (74%) BP profiles and increases the risk of non-dipping by ~47% [26,55,56]. Similarly, patients with non-dipping or reverse-dipping patterns show a 2.7-fold or 3.5 fold higher risk of developing OSA than those with normal dipping [56].

Beyond overall AHI, the distribution of respiratory events across sleep stages may also influence cardiovascular risk. In particular, REM (rapid eye movement)-predominant OSA has gained attention because obstructive events during REM sleep are often longer, associated with deeper oxygen desaturation, and occur during a phase characterized by greater autonomic instability and sympathetic activation. These mechanisms may translate into a stronger hypertensive burden than events occurring predominantly during non-REM sleep. Observational data suggest that an elevated REM-AHI is associated with both prevalent and incident hypertension, even in some patients with low overall AHI [57]. Therefore, REM-specific respiratory burden may provide additional prognostic information beyond conventional AHI and could help identify patients who remain clinically relevant despite apparently mild disease. However, diagnostic criteria remain heterogeneous, prevalence varies according to the definition used, and evidence regarding long-term cardiovascular prognosis and optimal treatment is still limited. Similarly, whether REM-predominant OSA requires specific therapeutic strategies beyond standard OSA management remains uncertain [58].

Despite a clear mechanistic link between OSA and hypertension, randomized trials in asymptomatic (i.e., without daytime sleepiness) patients have not shown a preventive effect of continuous positive airway pressure (CPAP) on incident hypertension or cardiovascular events over long-term follow-up [59]. Conversely, observational data indicate that CPAP may lower the risk of developing hypertension in previously normotensive patients with OSA [60,61,62]. Two meta-analyses conducted on patients with resistant hypertension highlighted the modest action of CPAP in reducing 24 h ambulatory levels of systolic (SBP, −5 mmHg) and diastolic (DBP, −3 mmHg) blood pressure, although with high interindividual variability [63,64]. Other systematic reviews further confirmed that CPAP use reduces 24 h BP (mean difference for SBP: 4.7–7.2 mmHg, DBP: 2.9–4.9 mmHg) and resting heart rate, especially among patients with severe OSA, high cardiovascular risk or resistant hypertension and high adherence to CPAP [64,65,66,67]. Of note, the impact of CPAP on BP is characterized by high interpatient variability. Therefore, CPAP lowers BP in resistant hypertension, but its role in primary prevention among asymptomatic OSA remains unproven.

### 6.2. Pulmonary Hypertension

PH is detected in 70–80% of OSA patients evaluated by right heart catheterization [68], and ~20% of all patients with OSA develop PH, which is associated with worse outcomes [69]. Intermittent hypoxia promotes pulmonary vasoconstriction, inflammation, and vascular remodelling, increasing pulmonary vascular resistance [70]. T < 90% was significantly associated with right ventricular systolic pressure, ejection fraction, hypertrophy and mean pulmonary artery pressure [71]. Patients with OSA and PH have higher mortality than those without PH [72]. CPAP therapy has been demonstrated to reduce mean pulmonary artery pressure by 5 mmHg after 12 weeks [73].

### 6.3. Coronary Artery Disease

OSA is an independent risk factor for CAD. Intermittent hypoxia–reoxygenation increases oxidative stress, inflammation, and endothelial dysfunction, promoting atherosclerosis and acute coronary events. Indeed, OSA is associated with increased arterial stiffness and early atherosclerosis [1,74]. Sympathetic activation in OSA increases endothelial adhesion molecules and leukocyte recruitment, contributing to plaque progression and instability [75,76]. An observational cohort showed a 2.5-fold higher risk of myocardial infarction (MI), coronary revascularization, or CV death in patients with OSA compared with controls [77]. Severe OSA is present in ~40% of patients presenting with ST-elevation MI [78]. Notably, patients with OSA have a higher incidence of nocturnal MI, likely driven by hypoxic and hemodynamic stress [79,80]. The impact of CPAP on prognosis, cardiovascular events, and revascularization procedures remains under debate.

### 6.4. Atrial Fibrillation

OSA is a major risk factor for AF, with SDB detected in up to ~70% of AF patients [81,82]. Nocturnal AF is more frequent in patients with OSA than in the general population (3–5% vs. 0.4–1%) [83,84]. Autonomic imbalance, atrial remodelling, and conduction abnormalities contribute to AF development in OSA [18,85]. Additionally, sympathetic surges and intrathoracic pressure swings promote atrial stretch and electrical instability [81]. The VARIOSA-AF study showed a night-to-night association between OSA severity and AF episodes [86]. In addition, severe OSA is linked to higher stroke risk and reduced efficacy of antiarrhythmic drugs and AF ablation [81,87,88]. Indeed, observational studies suggest that patients with OSA have a ~30% higher risk of post-ablation AF [89]. In addition, patients with high-frequency AF (defined as persistent or paroxysmal with >6 symptomatic episodes of AF in the previous year) have an enhanced prevalence of SDB than those with low-frequency AF (paroxysmal with <6 symptomatic episodes in the previous year) [90]. Observational studies and recent meta-analyses have shown that CPAP reduces AF recurrence after cardioversion or ablation [91,92,93,94], whereas non-administration of CPAP increases recurrence risk by 57% [89]. Lifestyle interventions (weight loss, alcohol restriction) also reduce AF relapse [95,96]. However, the SAVE trial did not show AF prevention with CPAP in patients with moderate or severe OSA with a history of ischemic heart disease or cerebrovascular disease, likely due to poor adherence (3.3 h per night), and the possible inclusion of patients with CA as the prevailing phenotype [97].

### 6.5. Other Arrhythmias

Patients with OSA show a higher prevalence of bradyarrhythmias [98,99], and in one polysomnography study, 58% of patients with pacemakers for sick sinus syndrome had previously unrecognized sleep apneas [100]. CPAP therapy is associated with a 72–89% reduction in bradyarrhythmia episodes, especially for nocturnal episodes, thus potentially reducing the need for cardiac implantable devices [99,101]. Severe OSA is linked to an increased risk of sudden cardiac death (SCD) [102], likely due to intrathoracic pressure swings and autonomic imbalance, which promote ventricular repolarization instability [103]. Ventricular tachycardia is more frequent in OSA than in non-OSA patients (2.24% vs. 1.16%) [104], and SCD occurs more often during the night in OSA (46% vs. 21%), with higher AHI predicting SCD between midnight and 6 a.m. [105]. The risk of non-sustained ventricular tachycardia is 3-fold higher in severe OSA compared to subjects without SDB, with a dose–response relationship between AHI and ventricular arrhythmia burden [106].

### 6.6. Heart Failure

OSA-related pathophysiological alterations worsen HF through neurohormonal activation, oxidative and inflammatory stress, BP surges, arrhythmias, and acute preload/afterload changes induced by intrathoracic pressure swings [18]. In HF, venous congestion and fluid retention promote an upper-body fluid shift in the supine position, increasing neck and lung pressure and favouring upper-airway collapse and OSA development [107]. SDB is highly prevalent in HF, affecting 50–75% of patients [108,109]. OSA and CA frequently coexist, although one phenotype is usually predominant [108]. In HF with reduced ejection fraction (HFrEF), patients with an OSA-predominant pattern show better biventricular function, exercise tolerance, and survival than those with CA-predominant breathing [3,110,111]. Apnea prevalence varies by HF phenotype: CA is more frequent in HFrEF than in HF with mildly reduced (HFmrEF) and preserved EF (HFpEF) (66% vs. 48% vs. 34%), whereas OSA is more prevalent in HFpEF than in HFmrEF and HFrEF (53% vs. 29% vs. 20%). Moderate-to-severe OSA is also more frequent in HFpEF (30% vs. 11% vs. 10%) [112,113]. SDB is an independent predictor of mortality, hospitalization, and cardiovascular events in HF [109]. In fact, severe OSA increases the risk of hospitalization by ~1.5-fold [114]. Recent studies suggest that CPAP therapy results in improved management of clinical resources (i.e., emergency room access, hospitalization) in patients with HF [115,116]. Randomized trials show CPAP reduces neurohormonal activation, arrhythmic burden, and echocardiographic parameters in HFrEF with sleep apnea [117,118], but no major trial has yet demonstrated a prognostic benefit on hard outcomes in HF patients with OSA [71,119].

Adaptive servo-ventilation (ASV) is an advanced positive airway pressure modality mainly used for CA and Cheyne–Stokes respiration, as it dynamically adjusts ventilatory support and can also treat coexisting obstructive events. The ADVENT-HF trial evaluated the effect of ASV in patients with HFrEF and SDB (OSA: 73%); ASV did not impact the composite endpoint of mortality, cardiovascular hospitalization, new-onset AF or flutter, and appropriate cardioverter–defibrillator shock. However, a positive difference was reported for quality of sleep and quality of life in the intervention group [120]. Although ASV may improve symptoms, the SERVE-HF trial showed increased cardiovascular and all-cause mortality in patients with symptomatic HFrEF and predominant CA who were treated with ASV [121]. Therefore, ASV is generally contraindicated in this specific setting. The apparently divergent findings of SERVE-HF and ADVENT-HF may reflect differences in patient selection, SDB phenotype, device algorithms, and treatment adherence, underscoring the need for careful phenotyping before considering ASV in HF. Further studies are needed to better define whether selected HF phenotypes may benefit from individualized use of ventilatory support [122].

## 7. Prognosis

Several studies confirm the negative prognostic impact of OSA on cardiovascular outcomes. In the MESA (Multi-Ethnic Study of Atherosclerosis) study, which involved 5338 patients without prior cardiovascular disease with a mean follow-up of 7.5 years, OSA predicted all-cause mortality (hazard ratio [HR]: 2.44, 95% CI: 1.36–4.37) and cardiovascular events, including MI, stroke, angina, resuscitated arrest, and cardiovascular death (HR: 2.16, 95% CI: 1.30–3.58) [123]. Other studies have shown that untreated severe OSA is associated with a ~3-fold increase in all-cause mortality compared with controls [61,124,125]. A meta-analysis of 16 studies (24,308 patients) confirmed higher overall and cardiovascular mortality in severe OSA compared with milder disease [126].

Sex-specific prognostic differences have also been observed. In a pooled analysis from ARIC (Atherosclerosis Risk in Communities) and SHHS (Sleep Heart Health Study) studies, women with severe OSA had more comorbidities and a higher risk of incident HF, CAD, left ventricular hypertrophy, or death than men [127].

## 8. Treatment

The management of OSA currently focuses on modifiable risk factors and the use of CPAP. Emerging pharmacological therapies may complement this framework rather than replace established treatments [17]. In particular, anti-obesity agents such as GLP-1 receptor agonists may be especially valuable in overweight or obese patients with OSA, whereas novel anti-apneic compounds may represent alternatives or adjuncts in selected patients intolerant to CPAP or with specific pathophysiological phenotypes. However, their role within clinical practice is still evolving, and further evidence is needed to define their long-term efficacy, safety, and optimal integration into treatment algorithms. Table 2 provides an overview of the currently available therapeutic options for obstructive sleep apnea.

### 8.1. Lifestyle Changes

Lifestyle change is the cornerstone of OSA treatment and includes abstinence from alcohol, adoption of an appropriate sleeping position, exercise, and weight loss through a hypocaloric diet. In patients with predominantly positional OSA, namely those with an AHI that decreases by ≥50% in the non-supine position, avoiding the supine position during sleep is recommended [128]. Several devices (e.g., structured pillows, positional supports, vibratory feedback systems) can promote lateral sleep [129]. A systematic review showed that positional therapy is superior to no intervention but less effective than CPAP [129].

### 8.2. Weight Loss: Lifestyle Interventions and Bariatric Surgery

Obesity is the main risk factor for the development of OSA and accounts for at least 40% of cases. It is therefore a crucial goal in the management of OSA [130]. Weight loss is always recommended for overweight or obese patients with OSA, even if asymptomatic or with mild symptoms [8,131,132]. Previous meta-analyses have demonstrated the beneficial impact of lifestyle interventions in this population in terms of reduction in AHI, ODI, ESS, and sleep-related parameters (i.e., arousal index and sleep efficiency), regardless of intervention duration or CPAP use [133,134,135,136,137,138,139]. Interestingly, lifestyle interventions displayed reduced effects in women compared with men, thus suggesting that OSA may present with a different phenotype in females [139]. The role of psychological or coaching support for weight loss still needs to be elucidated [140].

In the largest study evaluating lifestyle intervention in obese patients with OSA and diabetes (i.e., the AHEAD Study), diet and exercise resulted in a mean weight loss >10 kg and a reduction in AHI of 9.7 events/h after one year of follow-up, compared with education in diabetes management alone [141]. In addition, patients with moderate-to-severe OSA undergoing combined therapy with CPAP plus weight loss (defined as dietary intervention and exercise sessions) showed a greater reduction in systolic and mean BP values, compared to subjects receiving CPAP alone or weight loss alone [142]. Few studies have evaluated the long-term effects of lifestyle intervention. Beneficial effects may persist after 4 years despite a significant weight regain [143]. Benefits of lifestyle intervention were significant across OSA stages, but a stronger effect was reported in severe OSA [144,145].

In cohorts of patients eligible for bariatric surgery, the prevalence of OSA was estimated to be approximately 75%, with most of them being severe [8,146,147]. Meta-analyses demonstrated that weight loss induced by bariatric surgery led to a significant reduction in BMI and AHI, but also to an improvement in pulmonary or sleeping functional parameters (forced vital capacity, mean SaO_2_, nadir SaO_2_, sleep efficiency, N3-REM%) and patient-reported measures of sleep quality (i.e., ESS) [148,149]. Pooled data displayed a mean reduction in AHI of 19 events/h, a mean BMI reduction of 12 kg/m^2^ after surgery, and a mean RDI reduction of 34 events/h, with OSA remission rates around 65% at 1 year [150]. Although bariatric surgery is highly associated with improvement in respiratory and sleep parameters (~68%), the incidence of OSA regression is considered relatively low (~16%) [147,151].

An inverse dose-dependent association between physical activity and prevalence of OSA exists [152]. In randomized clinical trials including patients with moderate or severe OSA, exercise resulted in a reduction of 24–34% in the severity of OSA, even in the absence of significant changes in body weight [135]. The mechanisms of this weight-independent benefit are still unclear but may be related to the redistribution of body fat, reduced nocturnal rostral fluid shift from the legs, increased tone of the pharyngeal muscles, and improved quality of sleep [8,135,153]. Pooled data reported a mean reduction in AHI of 6–7 events/h, improved ESS score and peakVO_2_, despite poor changes in BMI [154,155,156,157]. Although CPAP was superior in determining AHI reduction compared to exercise alone in patients with HF and OSA, exercise was superior to CPAP in improving daytime sleepiness and quality of life, but the two interventions had a synergistic effect when combined [158]. Moreover, the combination of resistance training and aerobic exercise is more effective compared with aerobic training alone [155,159]. In addition to systemic exercise, one meta-analysis demonstrated that oropharyngeal or respiratory exercise (e.g., lip or breathing exercise) led to a reduction in snoring and a 50% decrease in AHI [160]. Although difficult to compare because of the heterogeneity in effect size and intervention procedures, aerobic exercise, combined exercise, and oropharyngeal exercise showed similar effects in patients with OSA [161].

### 8.3. Weight Loss: Pharmacological Therapy

Pharmacological therapy has gained an important role in achieving significant and sustained weight loss. In particular, the glucagon-like peptide-1 receptor agonists (GLP-1RAs), which were originally studied in the context of type 2 diabetes, have emerged as a novel class of pharmacological agents capable of pleiotropic effects by regulating glucose and lipid metabolism [162]. GLP-1RAs promote insulin secretion, inhibit glucagon release, delay gastric emptying, reduce central appetite drive, and exert systemic anti-inflammatory effects [163,164]. Clinical trials investigating GLP-1RA beyond diabetes have reported beneficial effects on weight loss, mortality, cardiovascular events, renal protection, and neurodegenerative outcomes across various populations, thus expanding the potential applications of these drugs [165,166,167].

The SCALE Sleep Apnea trial included obese patients with moderate-to-severe OSA who were unwilling or unable to use CPAP and were randomized to liraglutide or placebo as an adjunct to diet and exercise. Liraglutide, administered at a dose of 3.0 mg, led to a modest reduction in AHI (mean difference: 6.1 events/h), body weight (mean difference: 4.2%), glycated hemoglobin, and SBP after 32 weeks of treatment compared with placebo [168].

The effect of tirzepatide, a dual agonist targeting both GLP-1receptor and the glucose-dependent insulinotropic polypeptide receptor (GIP-R), has recently been investigated in the SURMOUNT-OSA trial [169,170]. The study included patients with OSA with or without concomitant CPAP therapy, randomized to tirzepatide or placebo. After 52 weeks of treatment with tirzepatide at a dose of 10–15 mg, patients achieved a weight reduction of 16% and 17% in the CPAP and non-CPAP groups, respectively. Compared with placebo, tirzepatide was associated with an additional AHI reduction of 20 and 24 events/h in patients with and without CPAP, respectively. In addition, tirzepatide reduced sleep apnea-specific hypoxic burden, calculated according to the frequency, duration, and depth of oxygen desaturation during polysomnography, compared with controls (mean difference: −70% min/h, and −61% min/h). Treatment with tirzepatide also reduced SBP, particularly among patients not receiving CPAP (mean difference: −7.6 mmHg, and −3.7 mmHg), as well as hs-CRP (mean difference: −0.7 mg/L, and −1.0 mg/L). Patient-reported sleep disturbance assessed using PROMIS-SRI and PROMIS-SD also improved with tirzepatide [170].

A recent meta-analysis of six studies, including patients with OSA (n = 1032) receiving GLP-1RAs, including tirzepatide or liraglutide, reported an estimated AHI reduction of 9.5 events/h compared with controls not receiving therapy [168,170,171,172,173,174,175,176]. Concomitant CPAP use did not appear to modify pharmacological effects [175].

Beyond weight loss, GLP-1RAs may also favourably affect OSA through remodelling of upper-airway fat deposition [165] and stabilization of respiratory drive and breathing rhythm [177].

### 8.4. Continuous Positive Airway Pressure

CPAP remains the first-line treatment for symptomatic OSA of any severity. By delivering continuous positive pressure through a nasal or oronasal mask, it prevents upper-airway collapse during sleep and normalizes AHI in most patients (>90%) [178,179,180]. Its effectiveness, however, is strongly dependent on adherence, with greater clinical benefit observed with longer nightly use. Although a minimum use of 4 h/night is often considered acceptable, higher adherence is consistently associated with better outcomes, and improved mask interfaces, behavioural support, and remote monitoring may enhance long-term use [8,178,179].

Beyond symptom control, the cardiovascular effects of CPAP have generated heterogeneous results. Overall, CPAP consistently lowers sympathetic activation and modestly reduces blood pressure, particularly in patients with resistant hypertension, severe OSA, or high cardiovascular risk. In selected cohorts, reductions in ambulatory BP of approximately 3–8 mmHg have been reported [63,64,65,66,67,181]. In patients with HFrEF and OSA, CPAP has also been associated with improvements in left ventricular ejection fraction, cardiac remodelling, pulmonary pressures, and right ventricular function [182,183], while observational studies suggest fewer hospitalizations and lower mortality [184,185,186].

In contrast, several large randomized trials evaluating CPAP for hard cardiovascular outcomes have been neutral. In the CERCAS trial, CPAP did not reduce incident hypertension or cardiovascular events in patients without prior cardiovascular disease, although *post hoc* analyses suggested benefit in adherent users (>4 h/night) [1,59]. Similarly, the SAVE trial, which enrolled patients with established cardio- or cerebrovascular disease, found no reduction in major cardiovascular events after 43 months despite improved sleepiness; however, patients with better adherence showed lower rates of stroke and cerebrovascular events [97]. Comparable findings emerged from the RICCADSA and ISAACC trials, in which overall event reduction was not demonstrated, whereas signals of benefit were more evident among patients with higher CPAP use or selected clinical profiles [187,188].

These apparently contradictory findings may be explained by several factors. First, many randomized trials excluded or underrepresented highly symptomatic patients, who may derive greater benefit from treatment. Second, average adherence in pragmatic trials was often modest and may have been insufficient to influence long-term cardiovascular risk. Third, cardiovascular benefit may require prolonged exposure and longer follow-up than that achieved in some studies. Finally, OSA is a heterogeneous disorder: patients with severe nocturnal hypoxemia, resistant hypertension, marked sleepiness, high hypoxic burden, or specific cardiometabolic phenotypes may respond differently than minimally symptomatic patients with milder disease.

This interpretation is supported by pooled analyses. Some meta-analyses focused on randomized trials reported no clear survival benefit [189], whereas larger analyses including observational cohorts have shown lower all-cause and cardiovascular mortality, particularly among adherent patients and those with moderate-to-severe OSA or concomitant CAD [66,190,191]. A dose–response relationship has also been suggested, with the greatest benefit in patients using CPAP for ≥6 h/night [66].

A particularly relevant unresolved issue concerns patients with asymptomatic or minimally symptomatic OSA. Current evidence does not support routine CPAP prescription solely for cardiovascular prevention in all such individuals, as major randomized trials largely failed to demonstrate a clear reduction in hard outcomes in these populations [97]. Nevertheless, selected asymptomatic patients with severe OSA, marked nocturnal hypoxemia, resistant hypertension, AF, or established cardiovascular disease may still derive benefit, particularly when adherence is high [187]. Therefore, treatment decisions in asymptomatic OSA should be individualized according to global cardiovascular risk, OSA phenotype, and patient preference.

Taken together, current evidence suggests that CPAP should not be viewed as a universal cardioprotective therapy for all patients with OSA. Rather, its prognostic benefit is likely concentrated in selected subgroups characterized by symptomatic disease, greater OSA burden, higher cardiovascular risk, and good treatment adherence. Future studies should focus on phenotype-guided patient selection and strategies to improve long-term adherence.

In addition to potential prognostic effects, CPAP consistently improves quality of life, daytime sleepiness, and functional status [180]. Benefits are greatest in patients with excessive daytime sleepiness or moderate-to-severe OSA, but can also be observed in selected patients with mild disease [192,193]. Meta-analyses further suggest modest improvements in general and sleep-related quality of life, cognition in older adults, and a marked reduction in road traffic accident risk [194,195,196,197].

Bilevel positive airway pressure (BiPAP), which provides inspiratory pressure support above expiratory positive airway pressure, may improve tolerance in patients intolerant to CPAP and is particularly useful in conditions associated with hypoventilation or hypercapnia (e.g., neuromuscular disease, chronic hypoventilation syndromes) [198,199].

### 8.5. Mandibular Advancement Devices and Surgical Procedures

Mandibular advancement devices (MADs) represent a non-invasive option for patients with mild–moderate OSA or for those intolerant to CPAP [8]. Patients with anatomical narrowing of the upper airways are likely to benefit most from MAD therapy. These devices consist of intraoral plates that advance the mandible, increasing upper-airway calibre. A meta-analysis of 34 studies showed a mean AHI reduction of 13.6 events/h (95% CI: 12.0–15.3) with MAD therapy [200]. Another meta-analysis of 51 studies reported comparable BP reductions with MAD and CPAP [201].

Surgery aims to enlarge or stabilize the upper airway but is supported by limited evidence, short follow-up, and scarce data on cardiovascular outcomes [202]. Uvulopalatopharyngoplasty reduces AHI but has largely been replaced by safer soft-tissue techniques (e.g., barbed and expansion pharyngoplasty) [203,204,205]. Maxillomandibular advancement is the most effective surgical option, reducing AHI by ~80% and improving ESS score (from 13.5 to 3.2) in selected patients intolerant to CPAP [206]. However, surgical morbidity (pain, malocclusion, aesthetic and sensory complications) limits its use, and long-term cost–benefit data remain insufficient [206,207].

### 8.6. Nerve Stimulation

Stimulation of upper-airway dilator muscles represents a novel therapeutic option for OSA. The only FDA-approved device delivers timed stimulation to the medial branch of the hypoglossal nerve via an implanted electrode, coupled with a thoracic sensor that detects respiratory effort and a subcutaneous pulse generator placed in the chest. This treatment was approved for patients unfit for CPAP with at least moderate OSA [208]. In the STAR trial, hypoglossal nerve stimulation reduced AHI from 29.3 to 9.0 events/h at one year, with sustained benefit at 5 years of follow-up [209,210]. Bilateral stimulation systems and non-invasive transcutaneous approaches are currently under evaluation, with preliminary results showing a good safety and efficacy profile [211,212,213]. A comparative study showed that hypoglossal stimulation improves daytime sleepiness to a greater extent than CPAP, while achieving a similar reduction in AHI [214].

### 8.7. Pharmacological Therapy

#### 8.7.1. Pharmacotherapy Targeting Pharyngeal Muscle Tone

Experimental pharmacological therapies for OSA primarily aim to enhance the tone of the upper-airway dilator muscles and improve central ventilatory control [215]. One approach involves modulating central respiratory control through drugs affecting noradrenergic and serotoninergic neurotransmission, referred to as “anti-apneic neuromuscular modulators.”

Among these, the combination of selective norepinephrine reuptake inhibitors (e.g., atomoxetine, reboxetine) and antimuscarinic agents (e.g., oxybutynin, hyoscine butylbromide) has shown reductions in AHI (35–62%) but failed to improve sleepiness [216,217,218,219,220]. Atomoxetine monotherapy has demonstrated inconsistent results regarding AHI and daytime sleepiness [220,221]. AD109, a combination of atomoxetine (75 mg) and oxybutynin (2.5 mg), has been tested in two Phase 3 trials [222,223]. In the SynAIRgy study (n = 639), AD109 significantly reduced AHI (6.1 vs. 0.4 events/h, *p* < 0.0001) and improved secondary endpoints like ODI and hypoxic burden. In the LunAIRo trial (n = 660), AD109 demonstrated a 46.8% reduction in AHI compared to 6.8% in the placebo group after 26 weeks, with improvements in hypoxic burden and ODI [224,225]. Although AD109 showed mild adverse effects, it demonstrated promising reductions in OSA severity, with 39.6% of participants experiencing a ≥50% AHI reduction [226].

On the other hand, the use of tricyclic antidepressants, such as protriptyline and desipramine, resulted in only modest reductions in AHI, with poor tolerability due to anticholinergic and cardiovascular side effects, making their routine use in OSA management questionable [227,228,229,230,231]. Similarly, selective serotonin reuptake inhibitors (SSRIs) like mirtazapine, paroxetine, and fluoxetine have shown inconsistent results in reducing AHI and are no longer considered viable options for OSA treatment due to limited clinical benefit and adverse effects [230,232]. The failure of SSRIs in OSA treatment may be linked to increased cholinergic inhibition of hypoglossal motor neurons during REM sleep, reducing their response to excitatory neurotransmitters [233]. Notably, mirtazapine was also associated with weight gain in one study [234].

#### 8.7.2. Pharmacotherapy Targeting Loop Gain

Certain forms of OSA are linked to altered acid–base balance due to increased chemoreceptor sensitivity, a condition known as “high loop gain” OSA [232,235]. High loop gain describes an unstable ventilatory control system, where small fluctuations in arterial CO_2_ or O_2_ levels trigger exaggerated ventilatory responses, leading to unstable breathing and promoting both OSA and CA [236]. Targeting the downregulation of high loop gain offers a potential pharmacological approach to OSA management.

Carbonic anhydrase inhibitors, such as acetazolamide, have been investigated as a potential treatment by reducing AHI through modulation of acid–base balance. However, clinical studies have shown mixed results. While acetazolamide has demonstrated effectiveness in CA, its impact on OSA has been less consistent, with meta-analyses reporting no significant reduction in AHI across studies [236,237]. The effects of acetazolamide in reducing AHI were modest, and further studies are required to better understand its role in OSA treatment.

A recent clinical study evaluated treatment for 4 weeks with the carbonic anhydrase inhibitor sulthiame (200 mg and 400 mg) in patients with moderate-to-severe OSA, reporting a reduction in AHI of 41% [238]. However, high dose sulthiame resulted in common paresthesias and headaches.

Other drugs, such as zonisamide and topiramate, have also been studied for their potential impact on OSA, but evidence of their effectiveness remains inconclusive. Zonisamide showed some benefit in reducing AHI and oxygen desaturation index (ODI), although adverse effects like dysphoria were noted in a proportion of patients [239].

Supplemental oxygen, another strategy aimed at modulating loop gain, has shown limited benefit compared to CPAP in reducing AHI and has not demonstrated improvements in blood pressure or sleepiness [240,241].

#### 8.7.3. Pharmacotherapy Targeting Arousal Threshold

Sedative and myorelaxant drugs, such as benzodiazepines and Z-drugs (zolpidem, zopiclone, eszopiclone), have traditionally been contraindicated in OSA due to concerns about worsening upper-airway collapse. However, recent studies suggest that these medications may have a beneficial effect in certain OSA patients, especially those with a low arousal threshold [232,242]. In these individuals, the intense ventilatory stimulus following an obstructive event may lead to early arousal, which disrupts breath and sleep stabilization. Sedatives could mitigate this issue by attenuating the arousal response, as suggested by studies in patients with moderate OSA [232,243].

The effectiveness of eszopiclone in reducing AHI remains controversial, likely due to study heterogeneity [244,245]. Additionally, the use of sedatives like temazepam has been associated with respiratory depression in OSA patients with high central chemosensitivity [243]. Other sedatives, such as trazodone and sodium oxybate, have shown mixed results in terms of AHI reduction, with limited evidence supporting their use for OSA treatment [232].

Dronabinol, a cannabinoid receptor agonist, has demonstrated some efficacy in reducing AHI and improving sleepiness in a small trial (PACE study), although its safety profile raises significant concerns, with neuropsychiatric and gastrointestinal side effects reported in 70–80% of participants [246,247]. Furthermore, the American Academy of Sleep Medicine does not recommend cannabinoids for routine OSA treatment due to insufficient long-term safety data [248]. While some trials suggest potential benefits of dronabinol in combination with other treatments like atomoxetine or acetazolamide, the overall evidence remains limited, and these therapies are not currently considered standard care for OSA.

#### 8.7.4. Wakefulness-Promoting Agents

An important consequence of OSA is excessive daytime sleepiness, which has a substantial impact on patients’ quality of life. A meta-analysis of eight studies demonstrated the efficacy of modafinil and armodafinil, dual dopamine-norepinephrine reuptake inhibitors, in improving sleepiness, attention and vigilance in patients with OSA, despite their lack of efficacy in improving quality of life and cognitive function [249]. However, these drugs may present contraindications due to their hypertensive and arrhythmogenic effects mediated by adrenergic activation [250]. Moreover, armodafinil led to weight loss because of its ability to suppress appetite when combined with lifestyle interventions over 6 months, although it did not improve OSA severity or sleepiness [251]. Modafinil and armodafinil have received FDA approval for patients with OSA and residual sleepiness.

A multicenter study including 244 patients with persistent OSA despite treatment with CPAP evaluated the effect of pitolisant, a histaminergic antagonist that promotes arousal in patients with OSA. The drug significantly reduced daytime sleepiness in treated patients compared with controls, despite being associated with more frequent adverse effects (mainly headache and insomnia) than placebo [252]. Finally, solriamfetol, a dopamine-norepinephrine reuptake inhibitor, has proven effective in improving sleepiness and quality of life in patients with OSA, despite a higher percentage of mild-to-moderate adverse effects (headache, nausea, loss of appetite, anxiety, nasopharyngitis) compared to placebo [253]. Systematic reviews confirmed the efficacy of wakefulness-promoting agents in improving self-reported sleepiness in patients with OSA, with solriamfetol being the most effective and pitolisant being the safest in patients with cardiovascular disease [254,255,256].

**Table 2:** Therapeutic options for obstructive sleep apnea.

Therapy (Drug/Device)	Mechanism of Action	Clinical Study	Clinical Outcomes	Clinical Indication
Non-Pharmacological Therapy				
**CPAP**	Provides continuous positive airway pressure preventing upper-airway collapse	MA: Benjafield et al., 2025 [66] MA: Feltner et al., 2022 [195] MA: Soltaninejad et al., 2025 [196] MA: Tregear et al., 2010 [197] MA [63,64,65,66,67] OS: Montesi et al., 2012 [73] OS [92,93,94] OS: Simantirakis et al., 2004 [98] RCTs [117,118,182] RCT: Barbe et al. [59]	↓Mortality and CV-Mortality ↑Sleep-QoL and overall QoL ↑Working memory and emotional scales ↓Road accidents ↓BP in resistant HTA ↓BP in PH ↓AF recurrence after DCCV/CA ↓Bradyarrhythmias ↓Neuro-hormonal activation, arrhythmias, ↑echo parameters in HFrEF No ↓of incident HTA	First-line therapy for symptomatic OSA
**Lifestyle** **Intervention for Weight Loss and Exercise**	Reduces peripharyngeal fat, fluid shift, and ventilatory load	MA [133,134,135,136,137,138,139,154,155,157] RCT: Chirinos et al., 2014 [142] RCT: Abed et al., 2016 [95]	↓AHI, ODI, ESS ↓BP ↓AF recurrence	Recommended in all overweight and obese patients
**Bariatric Surgery**	Marked weight loss and airway fat reduction	MA [148,149]	↓BMI, AHI, RDI, ESS, ↑Sleep-parameters	Recommended in selected obese patients
**MAD**	Mandibular protrusion enlarges upper-airway lumen	MA: Ramar et al., 2015 [200] MA: Bratton et al., 2015 [201]	↓AHI ↓BP similar to CPAP	Alternative to CPAP in mild–moderate OSA or CPAP intolerance
**Hypoglossal Nerve Stimulation**	Stimulates upper-airway dilator muscles	STAR trial [209] OS: Heiser et al., 2022 [214]	↓AHI, ODI, T < 90%, ESS, FOSQ scale ↑Effect size vs. CPAP	Approved for selected moderate–severe OSA unfit for CPAP
**Supplemental** **Oxygen**	Reduces hypoxemia and chemoreflex drive	MA [240,241]	Modest ↓AHI (inferior to CPAP) No effect on BP or sleepiness	Not recommended as monotherapy
**Pharmacological Therapy**				
**GLP-1RA**	Weight loss, reduced hypoxic burden and upper-airway fat through appetite suppression and metabolic regulation	SCALE Sleep Apnea trial [168] SURMOUNT-OSA trial [170] MA [175,176]	Liraglutide: ↓AHI, HbA1c, SBP Tirzepatide: ↓weight, AHI, hypoxic burden, SBP, hs-CRP, PROMIS-SRI, and PROMIS-SD scales ↓AHI	Approved for obesity. It represents an emerging option for OSA.
**Acetazolamide**	Carbonic anhydrase inhibition lowers loop gain	MA [237] RCT: Eskandari et al., 2018 [239]	Controversial results about AHI reduction Synergic effect with CPAP on BP and AHI	Although effective in CSA, experimental/selective role in OSA
**Sulthiame**		RCT: Hedner et al., 2022 [238]	↓AHI, high rate of AEs	Experimental, not guideline-recommended
**Zonisamide**		RCT: Eskandari et al., 2014 [239]	↓AHI High rate of AE	
**AD109** **(aroxybutynin +** **atomoxetine)**	Noradrenergic and antimuscarinic action increases tone of airway dilators	RCTs: LunAIRo and SynAIRgy trials [225,226]	↓AHI, ODI, PROMIS-Fatigue and Sleep Impairment T-scores	Experimental, not guideline-recommended
**TCA (protriptyline, desipramine)**	Noradrenergic action increases tone of airway dilators	RCTs [227,229] MA: AbdelFattah et al., 2020 [230]	Modest ↓AHI	Not recommended due to poor tolerability
**SSRIs (mirtazapine, paroxetine,** **fluoxetine)**	Serotonergic modulation of airway motor neurons	MA: AbdelFattah et al., 2020 [230]	Modest ↓AHI, No effects on sleepiness	Not recommended
**Z-drugs (eszoplicone, zolpidem), sedatives (trazodone, sodium oxybate), and** **pimavanserin**	Increases arousal threshold	RCTs: [244,245]	Controversial results about AHI reduction and sleepiness	Experimental; not guideline-recommended; safety concerns
**Dronabinol**	Cannabinoid receptor agonist modulating ventilatory control and arousal threshold	PACE trial MA [247]	↓AHI High rate of AE (70–80%) Effective but unsafe in combination therapy (acetazolamide, atomoxetine)	Not recommended by AASM [249]
**Modafinil/** **Armodafinil/** **Solriamfetol**	Dopamine– norepinephrine reuptake inhibition promoting wakefulness	MA [249,254,255,256] RCT [253]	↓Sleepiness, attention, and vigilance CV-AEs	Approved for residual sleepiness in treated OSA without CV contraindications
**Pitolisant**	Histamine H3 inverse agonist promoting wakefulness	MA [249,254,255,256] RCT [252]	↓Sleepiness AE: headache and insomnia	Approved for residual sleepiness in OSA

AE, adverse event; AF, atrial fibrillation; AHI, apnea–hypopnea index; BMI, body mass index; BP, blood pressure; CA, catheter ablation; CSA, central sleep apnea; CPAP, continuous positive airway pressure; DBP, diastolic blood pressure; DCCV, direct-current cardioversion; ESS, Epworth sleepiness scale; FOSQ, functional outcomes of sleep questionnaire; GLP-1RA, glucagon-like peptide-1 receptor agonist; HbA1c, glycated hemoglobin; HFrEF, heart failure with reduced ejection fraction; hs-CRP, high-sensitivity C-reactive protein; HTA, hypertension; IS, interventional study; MA, meta-analysis; ODI, oxygen desaturation index; OS, observational study; OSA, obstructive sleep apnea; PH, pulmonary hypertension; PROMIS, patient-reported outcomes measurement information system; PROMIS-SRI/PROMIS-SD, sleep-related impairment/sleep disturbance; QoL, quality of life; RCT, randomized controlled trial; RDI, respiratory disturbance index; SBP, systolic blood pressure; ↑, increased; ↓, decreased.

## 9. Who Should Be Screened and Treated for OSA?

**Figure 3:**
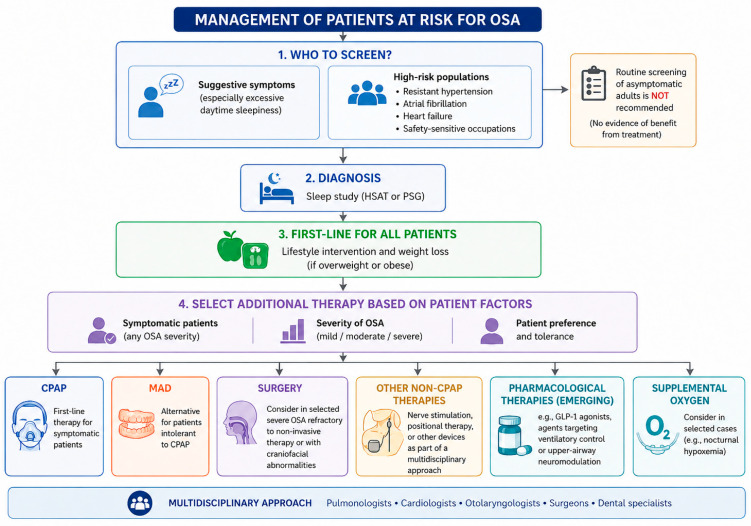
Proposed practical algorithm for screening and management of patients at risk for obstructive sleep apnea (OSA). HSAT: home sleep apnea testing; PSG: polysomnography; CPAP: continuous positive airway pressure; MAD: mandibular advancement device; GLP-1: glucagon-like peptide-1 receptor agonist.

There is no universal consensus on systematic screening strategies for OSA [43,131]. A practical approach to screening and management of patients at risk for OSA is summarized in Figure 3.

Routine screening of asymptomatic adults is not recommended by the U.S. Preventive Services Task Force or the American College of Physicians, given the lack of evidence that early detection and treatment in asymptomatic individuals improves clinical outcomes. Current recommendations support targeted screening in patients with suggestive symptoms, particularly excessive daytime sleepiness [257], and in selected high-risk populations, such as patients with resistant hypertension, AF, HF, or those working in safety-sensitive occupations [8,258]. However, the impact of screening for OSA on clinical outcomes is unclear, and no evidence currently supports treatment of asymptomatic patients with OSA for prognostic benefit [195].

Lifestyle intervention, especially weight loss in overweight or obese patients, is universally recommended across guidelines and should be considered first-line therapy irrespective of symptom burden. Beyond lifestyle measures, treatment selection is primarily guided by symptom severity, OSA burden, and patient preference [131,259,260]. In symptomatic patients, the American Heart Association and the American College of Cardiology recommend CPAP as first-line therapy, with MAD as an alternative for patients who are intolerant of CPAP [18,131]. Surgical options may be considered in selected cases of severe OSA refractory to non-invasive treatments or in patients with specific craniofacial abnormalities, although long-term cardiovascular outcome data remain limited, as highlighted by the American Academy of Sleep Medicine and the American Academy of Family Physicians [131,261]. Non-CPAP therapies are increasingly recognized as part of a multidisciplinary approach to OSA management. In this context, the European Respiratory Society emphasizes individualized treatment strategies involving pulmonologists, cardiologists, otolaryngologists, surgeons, and dental specialists [262]. Emerging therapeutic options, including pharmacological agents targeting obesity or ventilatory control and upper-airway neuromodulation techniques, offer promising alternatives but require further validation.

## 10. Conclusions

OSA is a common and often underdiagnosed condition with major cardiovascular implications. While treatment consistently improves symptoms and quality of life, evidence for cardiovascular event reduction remains heterogeneous and appears strongly influenced by patient selection and treatment adherence. Future research should focus on refining screening strategies, improving long-term adherence to therapy, and identifying subgroups of cardiovascular patients most likely to derive prognostic benefit from targeted OSA treatment.

## Figures and Tables

**Table 1:** Diagnostic tools for screening and diagnosis of obstructive sleep apneas.

Tool	Description	Advantages	Disadvantages
Questionnaire			
**Epworth Scale**	Assessment and staging of daytime sleepiness in different situations	Useful screening tool Accurate estimation of sleepiness Effective evaluation of therapeutic response	Nonspecific for OSA
**Berlin** **Questionnaire**	Assessment of the likelihood of OSA based on snoring, apneas, asthenia, sleepiness, obesity, hypertension	Useful screening tool Low score is associated with a low OSA risk	Low specificity in stratifying OSA severity
**STOP-Bang** **Questionnaire**	Assessment of the likelihood of OSA based on snoring, sleepiness, apneas, hypertension, obesity, neck width, age, sex	Useful screening tool Low score is associated with a low OSA risk	Low specificity in stratifying OSA severity
**Monitoring Systems**			
**Traditional** **Polysomnography**	Multi-channel recording (EEG, EOG, EMG, ECG, SaO_2_, thoracoabdominal movements, airflow)	Diagnostic accuracy Phases of sleep and arousals Current gold standard	High cost Low availability “First-night” effect Only at night
**Ambulatory** **Polysomnography**	Multi-channel recording (EEG, EOG, EMG, ECG, SaO_2_, thoracoabdominal movements, airflow)	Phases of sleep and arousals Ambulatory Reduced “first night” effect	High cost Reduced accuracy and validation Only at night
**Cardiorespiratory Monitoring**	Multi-channel recording (ECG, SaO_2_, thoracoabdominal movements, airflow)	Ambulatory Reduced discomfort Duration up to 24 h Low cost High availability	Absence of EEG Possible underestimation Reduced accuracy and validation
**Oximetry/airflow**	Mono-channel recording (SaO_2_ or airflow)	Ambulatory Reduced discomfort Low cost High availability	Absence of EEG Possible underestimation Not discriminating OSA and CA
**Implantable** **Devices**	Assessment of transthoracic impedance by sensors included in PM/ICD	Ambulatory Continuous recording Integration with different data (arrhythmias, congestion estimation, …)	Limited to PM/ICD carriers Intervendor variability Limited discrimination of OSA and CA Absence of EEG and SaO_2_

CA, central apneas; EEG, electroencephalogram; EMG, electromyogram; EOG, electrooculogram; ICD, implantable cardioverter–defibrillator; OSA, obstructive sleep apnea; PM, pacemaker; SaO_2_, oxygen saturation.

## Data Availability

Data sharing is not applicable to this article as no datasets were generated or analyzed during this current study.

## Abbreviations

The following abbreviations are used in this manuscript:

AF	Atrial fibrillation
AHI	Apnea–hypopnea index
BMI	Body mass index
CA	Central apnea
CAD	Coronary artery disease
CI	Confidence interval
CPAP	Continuous positive airway pressure
EEG	Electroencephalogram
ESS	Epworth sleepiness scale
GLP-1RA	Glucagon-like peptide-1 receptor agonists
HFr/mr/pEF	Heart failure with reduced/mildly reduced/preserved ejection fraction
HR	Hazard ratio
MAD	Mandibular advancement devices
MI	Myocardial infarction
ODI	Oxygen desaturation index
OSA	Obstructive sleep apneas
PH	Pulmonary hypertension
RDI	Respiratory disturbance index
REI	Respiratory event index
RR	Risk ratio
SAQLI	Calgary sleep apnea quality of life index
SaO_2_	Oxygen saturation
SCD	Sudden cardiac death
SDB	Sleep-disordered breathing
SF-36	Short Form-36 questionnaire
S/D-BP	Systolic/diastolic blood pressure
T < 90	Time spent with SaO_2_ < 90%
